# Comparison of whole blood and spleen transcriptional signatures over the course of an experimental malaria infection

**DOI:** 10.1038/s41598-019-52388-y

**Published:** 2019-11-01

**Authors:** Carlos Talavera-López, Yaw Bediako, Jing-wen Lin, John Joseph Valletta, Mario Recker, Jean Langhorne

**Affiliations:** 1 The Francis Crick Institute, London, United Kingdom; 2 West African Centre for Cell Biology of Infectious Pathogens, University of Ghana, Accra, Ghana; 3 Division of Pediatric Infectious Diseases, State Key Laboratory of Biotherapy, West China Second Hospital, Sichuan University and Collaboration Innovation Centre, Chengdu, 610041 China; 4 College of Engineering, Mathematics and Physical Sciences, University of Exeter, Exeter, United Kingdom

**Keywords:** Gene regulation in immune cells, Malaria

## Abstract

Although the spleen is broadly accepted as the major lymphoid organ involved in generating immune responses to the erythrocytic stages of the malaria parasite, *Plasmodium*, human splenic tissue is not readily available in most cases. As a result, most studies of malaria in humans rely on peripheral blood to assess cellular immune responses to malaria. The suitability of peripheral blood as a proxy for splenic immune responses is however unknown. Here, we have simultaneously analysed the transcriptomes of whole blood and spleen over 12 days of erythrocytic stage *Plasmodium chabaudi* infection in C57BL/6 mice. Using both unsupervised and directed approaches, we compared gene expression between blood and spleen over the course of infection. Taking advantage of publicly available datasets, we used machine learning approaches to infer cell proportions and cell-specific gene expression signatures from our whole tissue transcriptome data. Our findings demonstrate that spleen and blood are quite dissimilar, sharing only a small amount of transcriptional information between them, with transcriptional differences in both cellular composition and transcriptional activity. These results suggest that while blood transcriptome data may be useful in defining surrogate markers of protection and pathology, they should not be used to predict specific immune responses occurring in lymphoid organs.

## Introduction

Malaria represents a serious public health and economic problem and remains one of the leading causes of death in the developing world. After a number of years of apparent decline, the incidence of Malaria has begun to increase with over 200 million cases reported worldwide in 2017^1,2^. The eradication of malaria is a major public health goal and the development of an effective vaccine will be crucial to this effort. Although it is widely accepted that the majority of immune processes involved in the control of *Plasmodium* infection occur in the spleen^3,4^, studies requiring human splenic tissue are not practical. Instead, most studies rely on the detection of immune signatures in peripheral blood to evaluate the performance of vaccine candidates^5,6^, or to identify markers of protective immunity or pathology in humans^7–9^. Despite this obvious discrepancy and mounting evidence of tissue-specific differences in gene expression during immune responses^10,11^, there has not been a comprehensive analysis of simultaneous measurements from blood and spleen over the course of a *Plasmodium* infection to evaluate how similar or dissimilar the immune signatures in these two tissues are.

Recent advances in high throughput technologies have facilitated the quantification of whole transcriptomes for a number of different tissues^12^ and cells types^13^, and have revealed significant tissue-to-tissue genomic and transcriptional variation. Furthermore, high levels of within-tissue heterogeneity have been demonstrated by single cell sequencing of multiple tissues^14^, and related to differences in biological behaviour in health and disease^15,16^. It is therefore important to evaluate how closely transcriptional measurements from peripheral blood reflect the immune processes occurring in the spleen.

One concern when using blood transcriptomics as a surrogate to measure host immune responses taking place in the lymphoid organs such as the spleen, is that blood and lymphoid organs have different cellular composition and it is difficult to determine whether the transcriptomic differences are due to this, or to different transcriptional profiles within cell populations. Furthermore, whole blood transcriptomics does not provide information on the transcriptomes of individual immune cell populations. For studies in experimental models such as mice, it is possible to isolate and compare individual cell populations from different tissues directly. However, for human studies this is not often possible, as in most cases, (especially those involving young children), the volumes of extracted blood preclude isolation of individual cell populations.

In order to investigate the utility and relevance of using transcriptomic signatures in blood as a proxy for immune responses in lymphoid tissue, we have used a mouse model of blood-stage malaria. We have compared the transcriptomic profiles of whole blood and of spleen and used computational deconvolution to determine cell-specific transcriptomic signatures. Specifically, we have used the malaria model of *Plasmodium chabaudi* infection in C57BL/6 mice to compare the transcriptomes of whole blood and spleen during first 12 days of an erythrocytic-stage infection using mouse microarrays. To this end, we used an integrative approach applying statistical learning methods to analyse these transcriptomes, exploring the day-to-day transcriptional changes occurring in each tissue, allowing us to distinguish between conserved and tissue-specific immune responses to infection. We also inferred the cellular composition of each tissue, allowing us to monitor the signature of transcriptional activity of individual immune cell populations within blood or spleen.

## Results

### Transcriptome analysis reveals distinct responses to acute blood-stage *P. chabaudi* infection in blood and spleen

The acute blood-stage infection of *Plasmodium chabaudi chabaudi* AS (PcAS) from which these blood and spleen samples were taken is described in detail in a previous publication^17^. Briefly, after inoculation of 10^5^ PcAS-infected red blood cells (iRBC) parasites were detected within four days in peripheral blood, reaching a peak parasitemia of approximately 25% iRBC eight days after initiation of infection; thereafter, parasitemia was controlled to subpatent levels by day 20. To investigate whether blood and spleen demonstrated similar or different immune responses during the acute phase of the infection, we performed a simultaneous transcriptome analysis of both tissues at days 2, 4, 6, 8, 10 and 12 post-infection (d2, d4, d6, d8, d10 and d12 respectively). Age-matched naïve samples collected at d0 and d12 were used as naïve controls to exclude transcriptional changes due to time.

#### Unsupervised analysis of differentially expressed genes in spleen and blood

**Figure 1:**
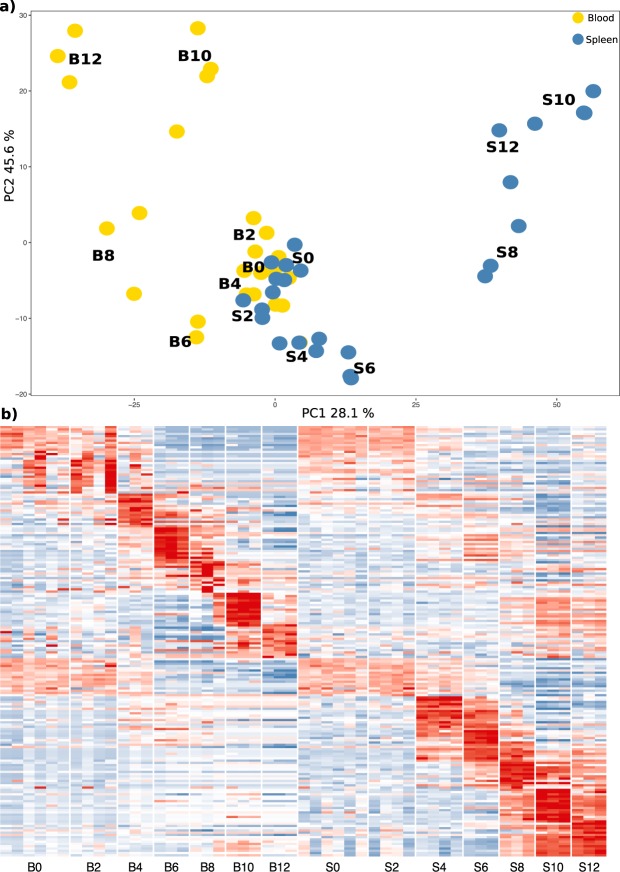
Merged expression matrix analysis shows distinct transcriptional patterns in blood and spleen over the course of acute *P. chabaudi* infection. (**a**) Principal component analysis of gene expression values from blood (yellow) and spleen (blue). (**b**) Gene expression signatures between the two tissues (B = blood, S = spleen) in the time-series composed of the top 10 significant differentially expressed genes (power > 0.7; −1.5 < logFC > 1.5) from the merged expression matrix. Colour scale represents expression value Z scores.

Separate analyses of blood and spleen transcriptomes do not readily allow for identification of genes and pathways that are shared between the two tissues. Therefore, we generated a merged normalised expression matrix for both tissues that allowed us to more directly compare the transcriptomic profiles of the two organs. Principal component analysis (PCA) reveals a distinctive distribution of data points for blood and spleen (Fig. 1a). Blood samples from naïve mice and from d4 are closer to each other than the same time points from spleen, suggesting that the spleen has an earlier transcriptional response to the infection. Later time points show divergence in opposite directions, indicating that these tissues feature distinct transcriptional programs during *P. chabaudi* infection. Differential expression analysis of the merged datasets comparing each time-point against their respective naïve control, identified 2975 significant DEGs (Area under the curve (AUC) > 0.85 and a logFC > 2) with each tissue presenting its own day-to-day signature (Fig. 1b and Supplementary File 1). Notably, in this merged analysis we noticed that while the spleen presents a distinct transcriptional pattern not present in the blood, it also mirrored part of the transcriptional signature observed in blood but at a lower expression level.

#### Pathway analysis of differentially expressed genes in spleen and blood

**Figure 2:**
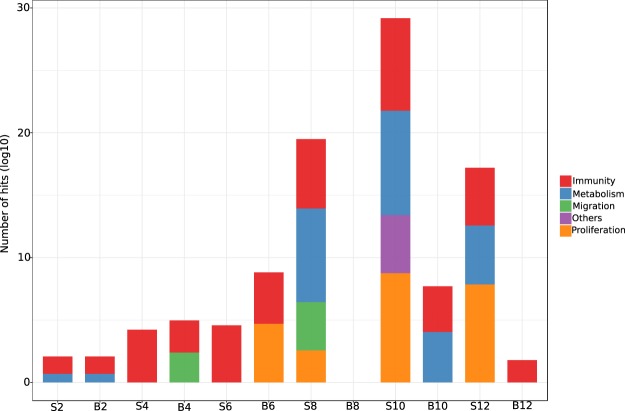
Pathway analysis of transcriptional signatures in blood and spleen over course of *P. chabaudi* infection. Differentially expressed genes (DEG) were queried against the Reactome pathways and those pathways with hits (number of DEGs matching the number of genes in a given pathway) that were significant (Benjamini-Hochberg *q-value* < 1e-5) were grouped into four major categories based on the nature of the biological process and those pathways with ambiguous classification were grouped under “*Others*”.

We next explored the functions ascribed to the DEG for each time point. DEG for each specific time-point in both tissues were then assigned to Reactome pathways using ToppFun^18^. Although 2975 DEG were identified, only 1151 DEG could be assigned to gene ontology (GO) categories or into biological pathways because of a lack of statistical support for goodness-of-fit into a given process, or because some of the microarray gene identifiers could not be found in current mouse genome annotations. The 1151 DEG were entered into the Reactome biological pathways module implemented in ToppFun and those that were significant (Hits > 10, Benjamini-Hochberg corrected *q-value* < 1e-05) were selected for further analysis. Given the diversity of the identified pathways, we grouped them into four categories: “*Immunity*”, “*Proliferation*”, “*Migration*” and “*Metabolism*” and computed the number of hits within all the processes contained in each category. Those processes that did not present a clear definition in any of the four categories were grouped as “*Others*” (Fig. 2 and Supplementary File 2). Blood and spleen share these categories; however, the relative number of hits and temporal distribution within each tissue is different. For instance, the spleen exhibits a strong metabolic signature between d8 and d12, which is only detectable at d10 in the blood. Genes associated with “Migration” are observed at d4 in the blood but appear much later (d8) in the spleen, perhaps indicating trafficking cells from blood to the spleen. All time points, except d8 in blood (which had no significant associations with any biological pathways), present an “Immunity” signature, but as can be seen in Supplementary File 2, the gene content of the shared pathways in the two tissues is, to a large extent, distinct.

**Figure 3:**
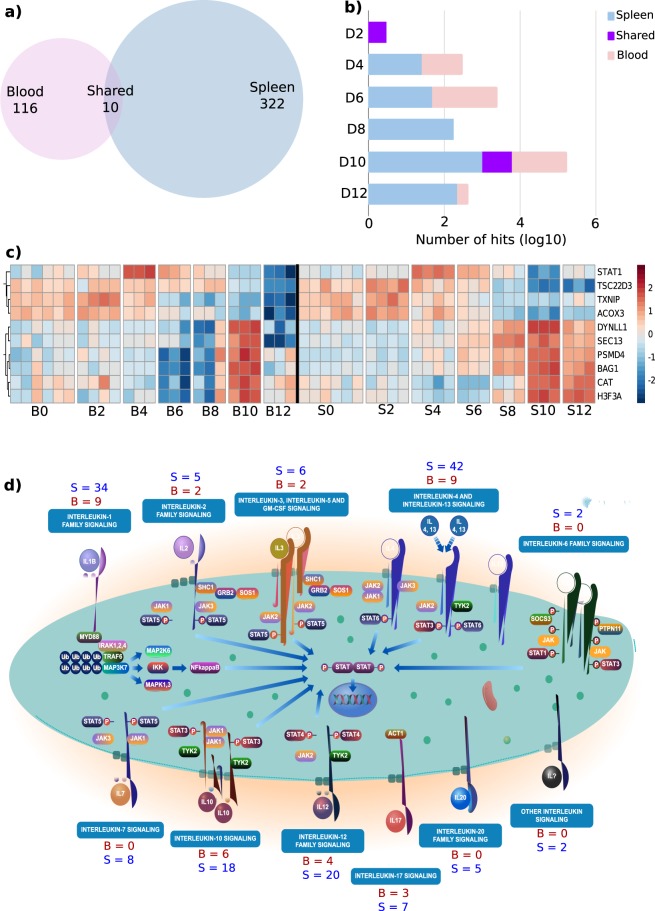
Distribution of shared and tissue-specific genes differentially expressed in blood and spleen over the course of *P. chabaudi* infection. (**a**) Number of tissue-specific (Blood = 116, Spleen = 322) and shared (10) immune-related DEGs. (**b**) Subclassification of tissue-specific and shared genes by day in log scale. (**c**) Heatmap of expression values (−2 < Z-score > 2) for the 10 immune-related genes shared between blood and spleen over the course of infection. (**d**) Reactome “Interleukin signalling” diagram showing the number of tissue-specific genes present in each process irrespective of the day of infection. Given the high level of diversity in the number of immune processes at each time point, Interleukin signalling was selected because it was the only process present at more than one time point (4 and 6 d.p.i) and had the highest significance.

Given the overlapping nature of the gene content within different pathways and by extension the four categories we created, we focused only on those genes related to the “*Immunity*” group and computed the non-redundant number of DEG that were allocated to each pathway at each time point for both tissues. We identified a total of 447 immune-related, non-redundant genes, 322 of these are unique to the spleen, 116 unique to blood, and only 10 genes are shared between both tissues at any time point (Fig. 3a and Supplementary File 3). We next asked how many of the genes were shared and how many were specific to spleen or blood on each day (Fig. 3b). At d2 there are only three genes, (*Acox3*, *Tsc22d3* and *Txnip*), that are shared. At d4, although both blood and spleen demonstrate increased levels of transcriptional activity compared to naïve controls, only *Stat1* is shared between the two. Overall, between d4 and d12, increasing numbers of “*Immunity*” genes are expressed in both tissues, but in general there are a greater number of genes expressed in the spleen. Only six genes (*Bag1*, *Cat*, *Dynll1*, *Psmd4* and *Sec*. 13) are shared at d10. The temporal expression dynamics of the immune-related genes shared between blood and spleen are detailed in Fig. 3c and it is interesting to note that despite the genes being shared, they demonstrate distinct patterns of expression over time in each tissue.

The diversity of pathways that were observed on each day meant that there were few of them present at more than one time-point. Within the non-redundant “*Immunity*” category, the “*Signalling by Interleukins*” pathway (as identified by Reactome) was the only pathway that was present at more than one time point during the infection, with sufficient significance at d2 (Hits = 20, Benjamini-Hochberg *q-value* = 6.61e-16) and d6 (Hits = 24, Benjamini-Hochberg *q-value* = 1.54e-11). Interleukin-signalling is essential for activation and regulation of the immune response and is therefore a good indicator of the host response to infection. At these two timepoints (d2 and d6), several genes within Interleukin-signalling pathways are represented in both spleen and blood (i.e. *IL-1*, *IL-2*, *lL-3*, *IL-4* and *13*, *IL-10*, *IL-12* and *IL-7*). In keeping with previous results, there are more genes differentially expressed in the spleen and despite being associated with the same signalling pathway, each tissue features a distinct set of genes. (Fig. 3d and Supplementary File 4).

#### Investigation of specific genes and/or pathways in spleen and blood

**Figure 4:**
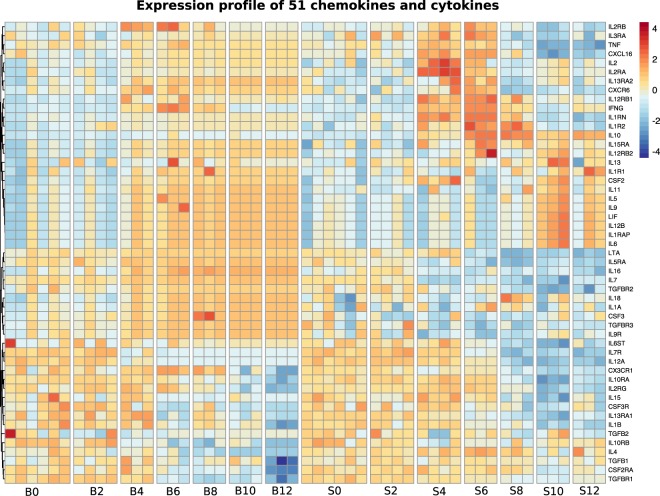
Directed comparison of cytokine expression in blood and spleen over the course of infection. A previously described^19^ set of 117 chemokines and cytokines was scanned against the merged microarray expression matrix and the resulting 51 genes were visualised based on their expression value in each tissue. Colour scale represents expression value Z scores.

Unsupervised analysis of differentially expressed genes in spleen and blood has shown that overall the transcriptome of blood and spleen before and during a *P. chabaudi* infection is different. It is however possible that a more directed analysis of particular molecules might indicate some common immune pathways. Given that our analysis identified “Signalling by interleukins” to be a significant component of both splenic and blood transcriptional responses to *P. chabaudi* we decided to employ a directed approach to compare cytokine responses within each tissue. We investigated the expression profile of a panel of chemokines and cytokines that has been previously described in both mice and humans^19^, comparing their relative expression in blood and spleen. From a total of 117 genes, encoding chemokines, cytokines or their receptors, we were able to identify 51 in each tissue microarray (Fig. 4). Interestingly, in agreement with the unsupervised analysis, we found that the expression profiles of these cytokines differed quite significantly between blood and spleen over the course of the *P. chabaudi* infection. The spleen exhibits a strong, progressive activation signature featuring many pro-inflammatory and Th-1 cytokines (i.e: *Tnf, IL-2, lL-10 and Ifng*) and chemokine receptors (i.e: *Cxcr6*, *Cxcl16*) beginning at d4 through d6, after which the signature appears to switch, with enhanced levels of Th2 cytokines (including *IL-13 IL-5* and *IL-9*). Blood on the other hand, demonstrates a weaker more heterogeneous activation signature from d4 that remains steady through the time points investigated. IL-10 is thought to be a key cytokine in regulating immunopathology in malaria^20^, and It is interesting to note that although *lL10* transcripts were expressed at high levels in the spleen from d4, they remained undetectable in the blood for the complete time course. Therefore, even this directed analysis of expression of specific immune molecules failed to reveal a significant number of genes shared between blood and spleen.

Overall our results show that the transcriptomes of whole blood and spleen over the 12 days of an erythrocytic infection of *P. chabaudi* are quite distinct. While there is some similarity in the immune pathways activated by the infection, the gene composition of these pathways is unique to each organ.

### Deconvolution of transcriptomic data reveals significant differences in cellular composition between spleen and blood

Blood and spleen differ quite significantly in terms of cellular composition^21^ and this influences their respective transcriptomic profiles. Our analyses of the DEGs of whole blood and spleen revealed many transcriptional differences with only few similarities between the two tissues, suggesting that one tissue was not an obvious surrogate for the other. However, our analysis up to this point does not account for the differences in cell composition between blood and spleen. As such we are unable to assess whether cell composition alone is responsible for the transcriptional profile differences between blood and spleen or if individual cell populations are also transcriptionally different between the two organs over the course of infection. The commonly accepted method for cell proportion estimation is flow cytometry, but it is not always possible to use this method in human field studies. For easier translation to the human setting, we therefore used a statistical learning approach using support vector regression to estimate the cellular proportions of immune cells in each tissue from the microarray expression data. We did this by building a mouse cell-specific transcriptional signature using publicly available transcriptome microarray datasets of purified immune cell populations from the Immunological Genome (ImmGen) project^22^ (See methods). This approach allowed us to define the proportions of several well-characterised immune cell types and compare the changes in cellular composition that occur in blood and spleen over the course of a *P. chabaudi* infection.

#### Determination of cell proportions in blood and spleen from transcriptome

We used the signature of 512 genes from the ImmuCC study^23^ to define our cell populations and we grouped them as follows: CD4^+^ T cells (CD4^+^ Naïve, CD4^+^ Memory, CD4^+^ Follicular, Th1, Th2 and Th17), CD8^+^ T cells (CD8^+^ Naïve, CD8^+^ Effector, CD8^+^ Memory), B cells (B Naïve, B Memory and Plasma cells), γδT cells, NK cells (Resting and activated NK), Myeloid cells (Macrophages, Monocytes and Dendritic cells (DCs)), granulocytes (Neutrophils, Eosinophils and Mast Cells).

**Figure 5:**
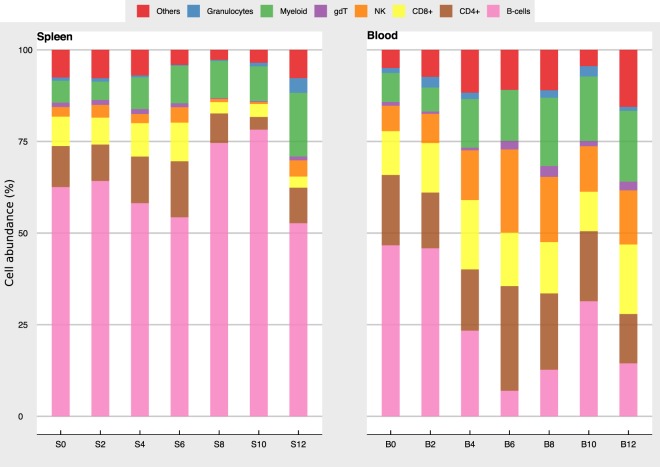
Inference of immune cell proportions from bulk microarray data. Each time-point was deconvolved into seven major immune cell populations using the ImmuCC signature. The myeloid group is composed of dendritic cells, macrophages and monocytes, while the granulocyte group is composed of Neutrophils, Eosinophils and Mast cells. Cells with proportions < 0.01% (T reg, Th1, Th2, Th17) were grouped into ‘Others’.

As infection with *P. chabaudi* changes the proportions of immune cell populations in different tissues^21^, we first evaluated the predictive accuracy of our estimates of cell proportions on uninfected C57BL/6 mice using the coefficient of determination (*r*^2^) and the root-mean-square error (RMSE) as metrics for cell classification and model accuracy. In this way we were able to assess how well our signature was differentiating each cell type (Supplementary File 5). We observed that the performance of the model was robust for both naïve spleen (**d0** *r*^2^ = 0.60, RMSE = 0.81) and naïve blood (**d0** *r*^2^ = 0.56, RMSE = 0.83) and performed reasonably well for the infected time points in spleen (**d2** *r*^2^ = 0.59, RMSE = 0.81, **d4** *r*^2^ = 0.45, RMSE = 0.89, **d6** *r*^2^ = 0.53, RMSE = 0.85, **d8** *r*^2^ = 0.79, RMSE = 0.72, **d10** *r*^2^ = 0.75, RMSE = 0.74, **d12** *r*^2^ = 0.44, RMSE = 0.90) and blood (**d2** *r*^2^ = 0.58, RMSE = 0.81, **d4** *r*^2^ = 0.56, RMSE = 0.82, **d6** *r*^2^ = 0.61, RMSE = 0.78, **d8** *r*^2^ = 0.56, RMSE = 0.82, **d10** *r*^2^ = 0.48, RMSE = 0.87, **d12** *r*^2^ = 0.52, RMSE = 0.85). We compared our cell proportion estimates with those already reported in the literature, and we observed good consistency between our predictions and flow cytometry estimates for immune cells available from the literature and from previously published studies from blood-stage *P. chabaudi* infections. For example, the observed proportion for B cells in naïve C57BL/6 spleen (S0) is estimated to be between 44–68%^21,24,25^ and our estimate is 62.5%. Similarly, our estimate is 46.6% in naïve blood (B0) is in line with published reports of blood B cell frequencies of between 35–58%. Estimates of smaller populations were also in agreement with expected frequencies. NK cells for instance have been reported to represent between 1–5% of splenic leukocyte populations and 4–7% of peripheral blood leukocytes in the mouse^25^ and our estimates for naive spleen and blood were between 2.6% and 6.9% respectively. Extending the analysis to include transcriptomic data from each organ over the course of infection, we found that the tissues showed marked differences in the proportions of the different cell populations during the first 12 days of the *P. chabaudi* infection (Fig. 5) as described previously for this infection using flow cytometry^21,24^. In the spleen, there was a reduction in the proportion of CD4^+^ T cells and an increase in B cells at days 8–10 post-infection as well as a marked increase in the proportion of myeloid cells at d12 post-infection. This expansion in the myeloid compartment has been observed previously in *P. chabaudi* infection and was found by us to be predominantly due to an increased number of monocytes^26^. The blood showed a substantial reduction in the proportion of B cells at days 6 and 8 post-infection with a proportional increase in NK cells and myeloid cells. Using computational methods, we have therefore quantified the cellular composition of blood and spleen and demonstrated that each tissue exhibits distinct cellular responses over the course of a *P. chabaudi* infection.

### Cell-specific transcriptional profiles describe the behaviour of each tissue in response to infection

Given the demonstrated differences in cellular composition between blood and spleen, it is necessary to assess differences in transcriptional activity between the two tissues independently of changes in cell number. To do this, we needed to shift from bulk whole-tissue analyses and instead compare the transcriptomic signatures of individual cell populations. Using the cell-specific signatures we defined above, we matched the differentially expressed genes from each tissue at any time point, to gene sets for each of the deconvolved populations using the ImmGen ‘*coexpression*’ module from ToppFun. Those that had a significant match (Hits > 20, Benjamini-Hochberg *q-value* < 1e-10) were further analysed over the course of infection (See methods).

**Figure 6:**
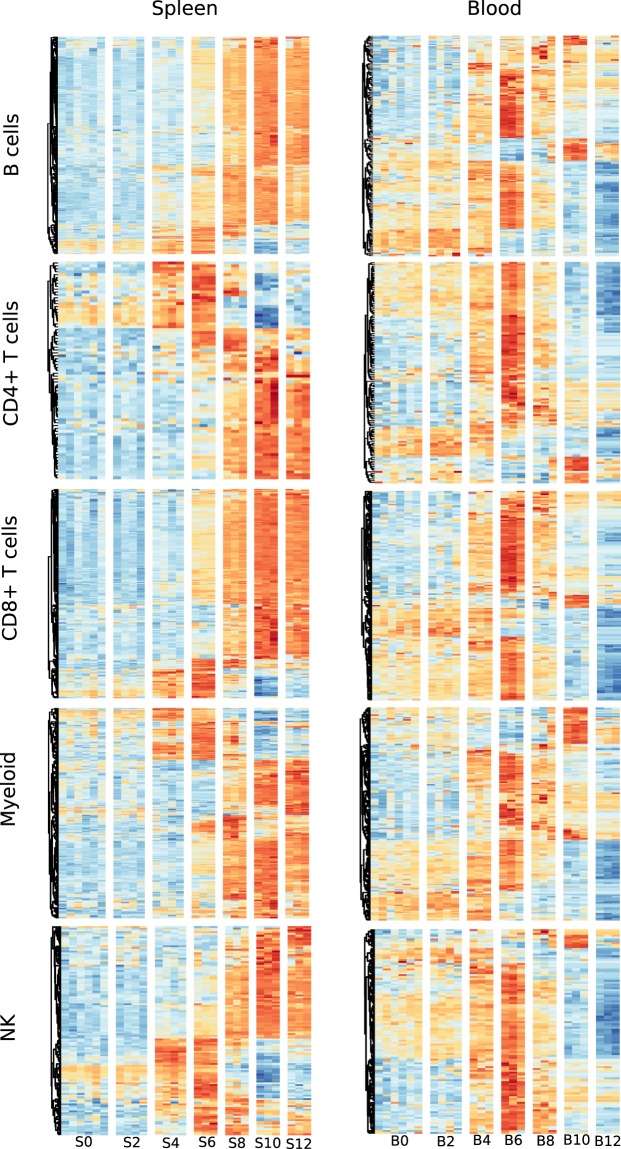
Comparison of cell-specific transcriptional signatures in blood and spleen over the course of infection. Differentially expressed genes (DEG) associated with five major cell types were visualised using heatmaps of their expression Z-scores (blue = −3, yellow = 0, red = +3). DEG from each tissue, irrespective of their time allocation were scanned against the ImmGen Phase 1 microarray signature and those with a match were used to build a heatmap and display temporal expression patterns.

Focusing on B cells, CD4^+^ T cells, CD8^+^ T cells, Myeloid cells and NK cells it is clear that each cell type expresses a distinct set of genes and expression profiles in each tissue over the course of infection (Supplementary File 6 and Fig. 6), possibly indicating that immune cell populations express unique tissue-specific transcriptional programmes. More specifically, most splenic cell populations show the first signs of activation on d4, upregulating a relatively restricted number of genes. Transcriptional activity continues to rise over subsequent days with a progressive increase in the number of genes upregulated up through d12. The same cell populations in the blood however appear to exhibit a different pattern of activation. There appears to be more baseline activity, with a number of genes already transcribed in naïve mice (d0), increasing gradually through d2 and 4, peaking at d6. In contrast to the spleen, the later time points feature significant downregulation of a large number of genes, starting on d8 and progressing through d12.

**Figure 7:**
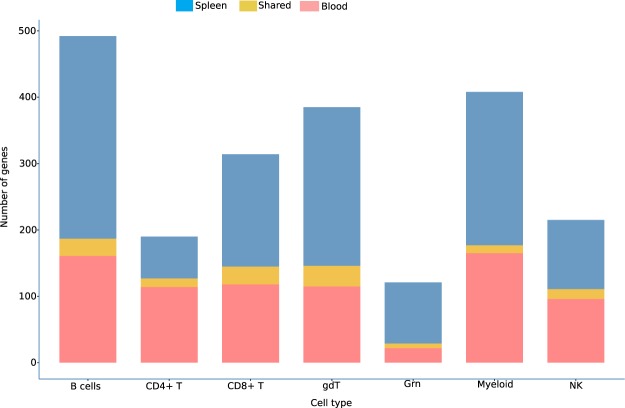
Distribution of shared and tissue-specific differentially expressed genes associated with each cell type. Number of differentially expressed genes associated with seven major immune cell populations expressed in either blood (pink) or spleen (blue). The number of genes that are expressed at one or more timepoints in both tissues is shown in yellow.

A closer look at these genes revealed that indeed very few genes are shared between the same cell populations from the two tissues. (Fig. 7 and Supplementary File 6). The majority of differentially expressed genes are ascribed to B cells (Spleen = 329, Blood = 185) and myeloid cells (Spleen = 242, Blood = 175), followed by CD8^+^ T cells (Spleen = 196, Blood = 145). We also observed a large fraction of genes associated with γδT cells (Spleen = 196, Blood = 145). Although the DE genes for each cell population in blood and spleen are largely mutually exclusive, only a small number of genes are shared between the two tissues for each cell type (Table 1 and Supplementary File 6), where *Asf1b, IL-18rap, Icos* and *Ifng* are the most shared genes between blood and spleen in multiple cell types. In summary, analysis of deconvolved cell–specific transcriptomes of the major immune cell populations in blood and spleen reveals very little similarity in gene expression between spleen and blood during *P. chabaudi* infection.

**Table 1:** Immune cell associated genes expressed in both blood and spleen over the course of infection.

B cells	CD4^+^ T cells	CD8^+^ T cells	γδ T cells	Granulocytes	Myeloid cells
*Agfg1*	*Agfg1*	*Asf1b*	*Asf1b*	*Creg1*	*Asns*
*Asf1b*	*Asns*	*Atp10a*	*Asns*	*Cyb5r1*	*Cox6a1*
*Asns*	*Cst7*	*Aurka*	*Aurka*	*Il18rap*	*Creg1*
*Aurka*	*Gata3*	*Aurkb*	*Aurkb*	*Lcn2*	*Cyb5r1*
*Aurkb*	*Gimap7*	*Birc5*	*Birc5*	*Myo1f*	*Lcn2*
*Birc5*	*Icos*	*Cdca2*	*Cdca2*	*Nqo1*	*Mthfd2*
*Cdca2*	*Il18rap*	*Cdca8*	*Cdca8*	*S100a9*	*Myo1f*
*Cdca8*	*Lgals1*	*Cenpa*	*Cenpa*		*Ndufa1*
*Cst7*	*Plk1*	*Crip2*	*Cox6a1*		*Pbk*
*Gata3*	*Rgs16*	*Cst7*	*Cox6b1*		*Plk1*
*Gimap7*	*Stat1*	*Gata3*	*Cst7*		*S100a9*
*Icos*	*Thy1*	*Gimap7*	*Gata3*		*Slamf8*
*Il18rap*	*Zap70*	*Icos*	*Gimap7*		
*Kif22*		*Ifng*	*Gstp1*		
*Lgals1*		*Il18rap*	*Icos*		
*Mcm3*		*Kif22*	*Igf2bp3*		
*Ncaph*		*Lgals1*	*Il18rap*		
*Ndufb6*		*Mcm3*	*Kif22*		
*Pbk*		*Myo1f*	*Lgals1*		
*Plk1*		*Ncaph*	*Mcm3*		
*Rgs16*		*Pbk*	*Ncaph*		
*Stat1*		*Pfkp*	*Ndufa1*		
*Sytl3*		*Plk1*	*Ndufb6*		
*Tcf19*		*Sytl3*	*Pbk*		
*Thy1*		*Tcf19*	*Plk1*		
*Zap70*		*Thy1*	*Rpa3*		
		*Zap70*	*Slc14a1*		
			*Sytl3*		
			*Tcf19*		
			*Thy1*		
			*Zap70*		

## Discussion

It is widely accepted that the main lymphoid organ involved in the immune response against blood-stage malaria parasites is the spleen^3,4^. However, since it is not feasible to obtain spleen biopsies for the purposes of studying malaria in humans, blood is routinely used (including in studies by the authors) as a surrogate to study the immune response to *Plasmodium* infection^27–31^. Furthermore, most studies in humans are cross-sectional and involve transcriptomic analysis carried out at a single time point, which only provides a snapshot of a dynamic and complex immune response.

Here we used the *Plasmodium chabaudi* mouse model of malaria and profiled both blood and spleen transcriptomes during an acute phase of infection, providing a unique opportunity to analyse and compare similarities and differences between the transcriptional responses occurring in each tissue over the course of the infection.

We found very few similarities between the transcriptomes of blood and spleen over the course of the infection. To identify shared genes or pathways better, we generated a merged normalised expression matrix for both tissues that allowed us to compare more directly the transcriptomic profiles of the two organs. In keeping with the results from the discrete analysis we found very little similarity between the transcriptomic profiles of blood and spleen. The two tissues did appear to share a number of immunological pathways, however closer analysis revealed that the gene content and expression kinetics of these pathways was tissue-specific. In an attempt to reveal any similarities between the two tissues, we conducted a directed analysis, comparing the levels of cytokine transcripts between spleen and blood over the course of the infection. Once again, spleen and blood were found to be quite distinct, with the spleen exhibiting a strong pro-inflammatory signature through d6 and then switching to a “Th2- type” response. The blood on the other hand had a much weaker and more heterogeneous expression profile that was maintained over the course of the infection. While some of the transcriptomic differences between blood and spleen can be attributed to differences in cellular composition, a comparison of cell-specific transcriptomic profiles, (generated through computational deconvolution) revealed significant differences in gene expression within the same major cell populations isolated from the two tissues.

One disadvantage of our study is the use of a microarray platform, which limits the scope of analyses that can be performed. However, for many crucial analyses, including cellular deconvolution which we perform in our study, microarray and RNA-Seq often yield very similar results. Indeed, using these microarray data we were able to identify differences in cellular proportions between the two tissues over the course of the infection to an acceptable level of accuracy. Ideally, the analysis of immune cell-specific signatures should be carried out using single cell RNA-Seq (scRNA-Seq) datasets from naïve and stimulated cells, since this is the most accurate way to know which genes are being expressed by which specific cellular population^32^. However, such analyses will not be feasible at the present time in the field for both practical and financial reasons. Our study provides evidence of the utility of using machine learning techniques together with comprehensive time-series transcriptomic data to facilitate the identification of important immune components during this rodent malaria infection.

Overall, our study indicates that blood and spleen have limited similarities during a *P. chabaudi* infection. This is an important observation as it suggests that information gained from signatures identified using whole blood transcriptomic analyses as a proxy for the response in the spleen (or other lymphoid tissue) as is done in many field studies, may be incomplete or even misleading. Given the impracticability of using human lymphoid tissue for most studies, we need to assess in which way blood transcriptomes are useful. Their value might lie in defining surrogate biomarkers of protection or immune pathology rather than in identifying specific components of the host response taking place in lymphoid organs.

## Methods

### Mice and parasites

All of the RNA samples described here were derived from a previously published experiment from our laboratory^17^. Briefly, female C57BL/6 aged 6–8 weeks from the SPF unit at the Francis Crick Institute Mill Hill Laboratory were housed under reverse light conditions (light 19.00–07.00, dark 07.00–19.00 GMT) at 20–22 °C, and had continuous access to mouse breeder diet and water. Mice were infected with a cloned line of *Plasmodium chabaudi chabaudi* AS, as described^33^. Infections were initiated by intraperitoneal (i.p.) injection of 10^5^ iRBC derived from cryopreserved stocks. Over the 12 days of the infection, the percentage of RBC infected (parasitemia) were monitored on Giemsa-stained thin blood films and the infection was as previously described for *P. chabaudi* AS^17^. All experimental work was carried out in accordance with the UK Animals (Scientific Procedures) Act 1986, under UK Home Office licence 80/2538 and 70/8326 to Jean Langhorne, and was approved by The Francis Crick Institute Ethical Committee.

### Infection and microarray data

Blood and spleens were collected at the same time from C57BL/6 mice that were intraperitoneally infected with 10^5^ *P. chabaudi* AS infected red blood cells at days 2, 4, 6, 8, 10 post-infection and from uninfected mice at times corresponding to days 0 and 12 of infection as described^17^. Total splenic RNA was extracted using RiboPure Kit (Ambion) following the manufacture’s protocol. cRNA samples were prepared from 300 µg globin reduced blood RNA or total splenic RNA using Illumina TotalPrep RNA Amplification Kit (Ambion) and hybridized to Illumina Mouse WG-6 v2.0 Beadarrays (consisting of 45,281 probe sets representing 30,854 genes) according to the manufacturer’s protocols. At each step, the quantity and quality of the RNA samples was verified using NanoDrop 1000 Spectrophotometer (Thermo Fisher Scientific) and Caliper LabChip GX (Caliper Life Sciences).

Blood transcriptomic data were previously published (GEO accession: GSE93631), and gene expression data from spleens were submitted under GEO accession number GSE123391.

### Microarray analysis

Microarrays were normalised using the *limma* package. The normalised expression matrix for each tissue was used as input for dimensionality reduction using Principal Component Analysis (PCA) with the *irlba* package in R using the *prcomp_irlba*() function to find approximate single vectors in a sparse matrix^34^.

For differential expression analysis we used the Standard AUC classifier implemented in the R *Seurat* toolkit^35^. In brief, for each gene in the expression matrix a classifier is built that will classify between two time-points and this classifier is evaluated with an Area Under the Curve (AUC). An AUC value of 1 means that the expression values of this gene alone can perfectly distinguish between the two groups being compared using the classifier, a value of 0.5 indicates that the gene cannot distinguish between the two groups being compared and a value of 0 indicates that the gene assumes that the two groups are the same.

The comparison was done in two steps, where expression values in each time point were first compared against their naive and those genes that were unique to the time point and had a classification power above 0.7 were designed as DEG between the time-point and its naive, then we took these DEGs and compare them against each time point in the time series until a set of unique DEG for that time point with a classification power above 0.85 and a logFC > 2 was selected. All the scripts for the analysis can be found on GitHub: https://github.com/cartal/spleen_vs_blood_microarray. DEGs were analysed functionally using the ToppFun tool from the ToppGene server as follows: We proceeded with these DEG and fit them into biological pathways that were significant (a hit number > 10 and a Bonferroni-Hochberg corrected *q-value* < 1e-5) using ToppFun, and the resulting pathways were grouped for simplicity into five main categories: *Metabolism*, *Proliferation*, *Migration*, *Immunity* and *Others*. Genes from the Reactome database that were associated with immune processes were compared against each other to identify tissue-specific and shared genes across the entire time-series.

### Characterisation of immune cellular proportions

To estimate the cellular proportions of immune cells in each tissue from the microarray data, we used the ImmuCC signature to infer mouse immune cell proportions^23^. We performed cellular deconvolution the ImmuCC signature with the support vector regression (SVR) implemented in CIBERSORT^36^, using 1000 permutations without quantile normalisation. Cellular proportions were accepted if they had a Pearson correlation coefficient r^2^ > 0.45 and a Root Mean Squared Error RMSE < 0.9 for the evaluation of the SVR model.

### Generation of an immune cell-specific transcriptional signature

We scanned the differentially expressed genes from each tissue irrespective of the time of infection against the characterised signatures of mouse immune cell types under different conditions from the ImmGen Stage 1 microarray dataset for cells isolated from lymphoid organs and blood as implemented in ToppFun (Supplementary File 7). Since these data contains signatures associated to the naïve and stimulated states of immune cells, it allows for the comprehensive characterisation of the mouse immune response. The output files from ToppFun were processed using a Python script (https://github.com/cartal/spleen_vs_blood_microarray) to select those signatures associated mouse immune cell populations that were significant (Hit > 20, Benjamini – Hochberg *q-value* < 1e-10). These genes were later used to create heatmaps to visualise their temporal behaviour.

## Supplementary information

Supplementary File 1

Supplementary File 2

Supplementary File 3

Supplementary File 4

Supplementary File 5

Supplementary File 6

Supplementary File 7

## Data Availability

The datasets generated during and/or analysed during the current study are available in github: https://github.com/cartal/spleen_vs_blood_microarray. Microarray data: MIAIME-compliant data are deposited in GEO: accession number GSE93631 (blood) https://www.ncbi.nlm.nih.gov/geo/query/acc.cgi?acc=GSE93631 and accession number GSE123391 (spleen).
